# Development and Face/Content Validation of the Care Load Scale Based on Hospitalized Patients’ Care Needs

**DOI:** 10.3390/nursrep15110380

**Published:** 2025-10-26

**Authors:** Alexander Casallas-Vega, Kevin Julian Aya-Roa, Judith Liliana Ortiz Mayorga, Lina Maria Vargas-Escobar, Marcia Andrea Quiñonez Mora, Genny Paola Fuentes Bermudez

**Affiliations:** 1 Faculty of Nursing, Universidad El Bosque, Bogotá 111321, Colombiagfuentesb@unbosque.edu.co (G.P.F.B.); 2 Faculty of Health Sciences, Nursing Program, Universidad de Cundinamarca, Girardot 252430, Colombia; kaya@ucundinamarca.edu.co; 3 Los Cobos Medical Center, Bogotá 110111, Colombia; jlortizm@loscobosmc.com (J.L.O.M.);

**Keywords:** nursing, nursing research, psychometrics, patient care planning

## Abstract

**Background/Objectives:** The burden of nursing care is defined as the relation between the care needs of hospitalized individuals and the time available for nursing staff to perform direct, indirect, and educational care activities. This study aimed to design an instrument to measure the burden of nursing care and to assess its face and content validity. **Methods:** This methodological study was conducted in three phases: (1) operationalization of the concept, (2) instrument design, and (3) face and content validity assessment. Expert panels using the nominal group technique were employed in phases one and two. In phase three, item evaluations regarding clarity, coherence, and relevance were conducted by experts. **Results:** Face validity was assessed by six expert researchers, while content validity was evaluated by 55 nurses with graduate-level education. The results demonstrated content validity index (CVI) values ranging from 0.89 to 0.95; Aiken’s V values between 0.84 and 0.94; and Kendall’s W concordance coefficients between 0.54 and 0.73, all statistically significant (*p* < 0.001). **Conclusions:** The Care Load Scale, designed to measure the burden of nursing care based on hospitalized patients’ needs, demonstrated strong face and content validity. The instrument shows potential for use in clinical settings to guide nursing care planning, allocate resources effectively, and inform institutional policies. The inclusion of expert judgment and rigorous validation procedures ensures the instrument’s relevance and applicability. This scale represents a significant contribution to nursing research and practice by offering a standardized tool aligned with patient-centered care principles.

## 1. Introduction

Globally, the nursing workforce is estimated to comprise approximately 28 million professionals, of whom 30% (8.4 million) are practicing in the Americas region [1]. Although nurses comprise 59% of the health workforce in this region (lower than the 69% reported worldwide), a worldwide shortage of 5.9 million nurses remains, according to recent data [1,2].

In Latin America, the average nurse staffing density between 2017 and 2021 was 44 nurses per 100,000 population [3]. In contrast, Colombia reported a density ranging from 10 to 19 nurses per 10,000 inhabitants in 2018, which rose to 68.7 per 10,000 inhabitants, totaling 66,095 registered nurses [2].

In addition to the nursing shortage in Latin America, various factors such as a lack of material resources, organizational conditions, institutional policies, clinical decision-making and the values and beliefs of the nursing team all contribute directly to an increased nursing care burden. This overload has two immediate, widely documented consequences: missed and rationed nursing care. These consequences affect health systems in all countries and at multiple levels of care [4,5,6]. Missed nursing care has been defined as the partial, delayed, or non-performance of necessary nursing interventions, and is considered a universal and concerning phenomenon in the scientific literature [7,8]. An exploratory review of 44 studies on unmet care needs from the patient’s perspective identified recurring failures in communication, emotional support, autonomy, self-care education, and psychological care activities [9]. An increase in omitted care and low staffing levels has been associated with lower patient safety levels, poorer care quality, lower job satisfaction, and a greater intention to leave the profession among nursing staff [10,11], as well as adverse events reported by patients and their perceptions of staff adequacy [12].

Nursing is distinguished as the health discipline with the most direct interaction with patients, placing it at the core of clinical care delivery [13]. At the heart of the nursing profession lies a commitment to addressing the care needs of individuals in order to restore health [14].

Meeting patients’ care needs is a key component of the burden of nursing care, which is defined as the relation between the needs of the subjects of care and the time required by nurses to conduct direct care, management, and educational activities [15].

In this sense, it is essential to have a measurement tool that assesses the care needs of hospitalized patients in order to quantify the burden of nursing care. Although several instruments exist to assess nurses’ workload, none have been identified that focus specifically on patient care needs as the basis for measuring nursing care burden [16].

Although complementary, nursing workload and nursing care burden are distinct concepts with different applications. Alghamdi [17] defines nursing workload as the total sum of activities performed by a nurse during their working day, including clinical, administrative, educational, and coordination tasks. It incorporates organizational, emotional and cognitive factors and is generally measured from an operational or institutional perspective. In contrast, nursing care burden focuses specifically on patients’ care needs and their direct impact on care planning. It is understood as a measure of the intensity of care required, expressed in terms of time and complexity based on clinical criteria including patient dependency, critical status, and required interventions [15]. While workload reflects the total responsibilities of nursing staff, care burden focuses solely on the care component related to the direct provision of care. This distinction is important because an overload of care can result in care being omitted or rationed, which affects the quality of care and patient safety. These issues are not always evident through traditional workload indicators.

From a nursing care perspective, patients are viewed as holistic beings, and their biological, psychological, sociocultural, and spiritual needs must be addressed comprehensively. This approach emphasizes that one of nursing professionals’ essential functions is to meet individuals’ multiple needs during their stay in healthcare institutions. According to Virginia Henderson’s theory, achieving or recovering health requires the continuous satisfaction of these needs, forming the basis of nursing practice and guiding interventions towards patient autonomy and well-being [18].

This study is theoretically founded on Virginia Henderson’s nursing theory, which conceptualizes nursing care as a comprehensive response to the individual needs of patients across biological, psychological, social, and spiritual dimensions. This holistic perspective highlights the importance of evaluating nursing care burden based not only on workload or staffing levels, but also on patient-centered needs [19,20,21].

When these needs are met through systematic, humanized care, people with illnesses are more likely to regain their health and well-being, achieving a state of physical and emotional balance [21]. This view enables us to distinguish clearly between the concepts of nursing care burden and nursing workload. The former encompasses the care needs of patients in all their complexity, including direct care, education and management activities, while the latter refers specifically to the quantification of the time (in hours or minutes) that a nurse requires to perform direct and indirect care activities with vulnerable individuals [22]. This distinction is essential to understanding the true scope of nursing work and its impact on the quality of care provided.

Although several instruments currently exist to measure nursing workload, such as the Nursing Activities Score (NAS), the Therapeutic Intervention Scoring System (TISS), and the RAFAELA system, these tools primarily quantify time or tasks related to direct care. They often omit contextual factors such as patient complexity, emotional care demands or educational interventions. Furthermore, many of these instruments are context-specific and oriented towards intensive care settings. They were also developed under managerial frameworks that focus on staff allocation rather than clinical reasoning [23,24]. Consequently, they fail to capture the multidimensional nature of nursing care as defined by patient needs. In contrast, the proposed scale integrates theoretical and clinical perspectives to offer a more comprehensive tool that reflects the actual care burden experienced by nurses in various hospital settings. This conceptual distinction justifies the development of a new instrument tailored to patient-centered care assessment.

Several studies have shown that a higher nursing care burden is associated with adverse events, nosocomial infections, hospital readmissions, and negative clinical outcomes [4,25,26]. The nurse–patient relationship is one of the elements present in the nursing care burden and is related to increased mortality rates, prolonged hospital stays, and higher readmission rates [27,28,29].

The physical and emotional demands of the hospital environment can affect nurses’ perception of their workload, thereby impacting their ability to provide safe, timely, and effective care [30]. Similarly, the time spent on clinical record-keeping and coordinating multiple tasks with other team members has been identified as significantly influencing the perceived workload [31]. Activities such as changing dressings, wound care, and hospital admission and discharge processes, as well as educational and administrative interventions, increase the time and resources required for each patient, thus increasing the care burden [32,33,34,35]. However, adequate resource management within the department can mitigate this burden by promoting collaboration and fostering teamwork [36].

The instrument developed to assess nursing care burdens is particularly relevant for nurse managers, as these professionals play a central role in planning, allocating resources, and making strategic decisions aimed at ensuring patient safety and the quality of care. Accurately quantifying patient care needs in context enables managers to identify imbalances between demand for care and staff availability, thus avoiding situations of overload that can lead to care being omitted, adverse events occurring, and user satisfaction decreasing. Such tools facilitate informed decision-making regarding staffing, work organization and care prioritization, all of which are key elements in promoting safe and efficient healthcare environments.

Therefore, it is necessary to design and validate an instrument grounded in theoretical nursing frameworks, integrating theory with practice and serving as a tool for guiding future research and decision-making. This study is part of a broader research project aimed at establishing the conceptual and instrumental foundations of the burden of nursing care. While the long-term objective of the project includes assessing the instrument’s construct validity through exploratory and confirmatory factor analysis, this article focuses specifically on the initial methodological phase: the design of the items and the validation of their content and appearance by a panel of experts. The objective of this phase is therefore to evaluate the methodological process of developing items and validating the content of a scale designed to assess the nursing care burden based on the care needs of hospitalized adult patients in internal medicine.

## 2. Materials and Methods

**Figure 1:**
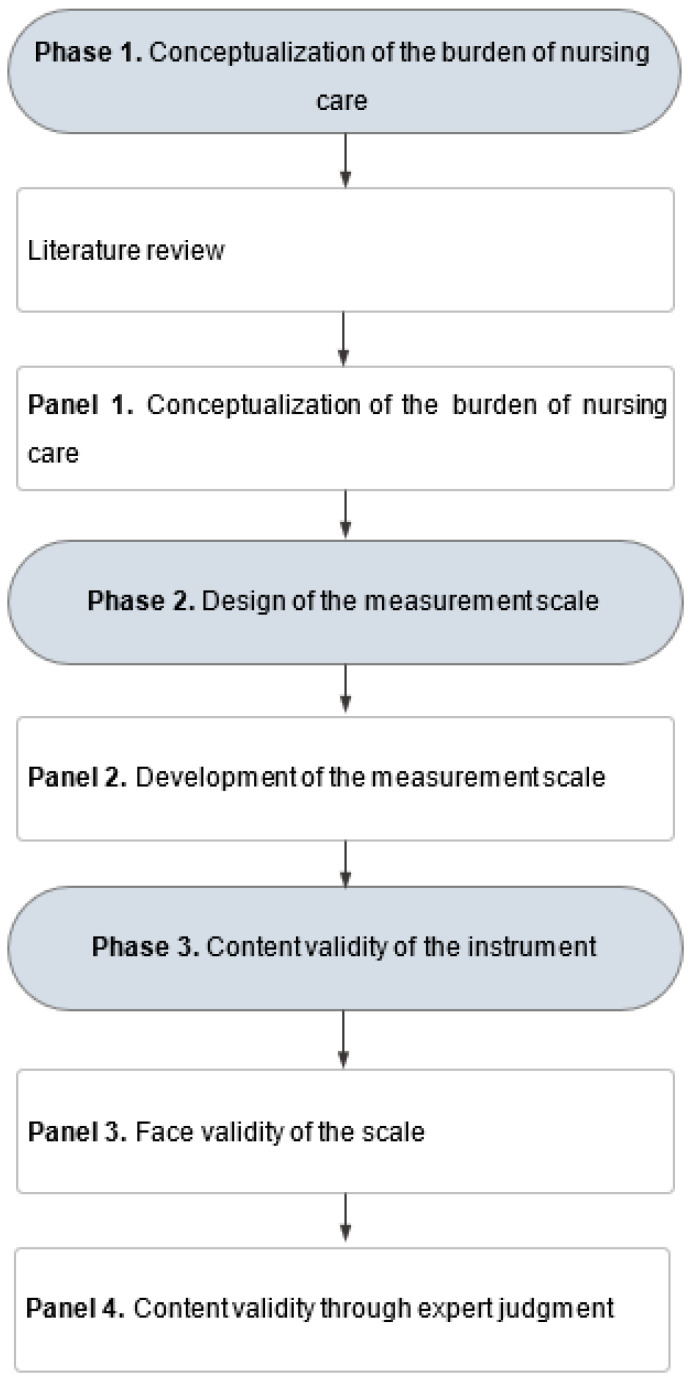
Methodological design.

This was a methodological study aimed at designing and validating the content, through expert judgment, of an instrument to measure the burden of nursing care, based on the framework proposed by Waltz [37]. The process was carried out in three stages, as illustrated in Figure 1.

### 2.1. Phase 1: Conceptualization of the Burden of Nursing Care

The conceptualization of the phenomenon represents the initial stage in the design and validation of this instrument. In this phase, a clear and precise definition of the variable was established by identifying its theoretical dimensions (care needs and time and resources available to nursing professionals). This process enabled the transformation of abstract aspects into empirically observable indicators [37]. To achieve this, a literature review was conducted, followed by the first expert panel using the nominal group technique [38].

Three working groups were held with the participation of different stakeholders in the health sector. The first group included three general directors of healthcare institutions. The second group consisted of four healthcare professionals with administrative responsibilities related to quality management at both local and national levels, as well as deputy finance directors. The third group consisted of four nursing directors from public and private health institutions of medium and high complexity.

During this first expert panel, the steps of the nominal group technique were followed: (1) silent generation, (2) round-robin sharing, (3) clarification, and (4) voting (ranking) [38]. In the silent generation phase, the research objectives and guiding questions were presented to each participant for individual reflection. This step lasted 15 min. In the second phase, participants engaged in a round-robin sharing session at each table, expressing their ideas and experiences. A designated recorder at each table documented every contribution and consolidated the ideas shared. There was no time limit for this phase.

The third phase, clarification, involved listening to the different positions on the questions posed in the previous phase and engaging in discussion within and between groups. This phase was also not time-restricted. The principal researcher moderated the academic discussion, while the other researchers compiled all the input. In the final step, all ideas were integrated, and a consensus was reached on the definition of the concept of nursing care burden. The guiding questions for the nominal group session were:

1. What is the nurses’ perception of the care provided in their institutions and Colombia more broadly?
2. What is the weight of the administrative burden in nursing activities?
3. How are internal (e.g., complexity, service openings, and closures) and external (e.g., policy changes) contextual changes incorporated into the allocation and distribution of nursing resources?
4. How can the burden of nursing care be defined?
5. What elements should a measurement instrument for the burden of nursing care include?

As a result of this first stage, nursing care burden was defined as the relation between the care needs of the subjects of care and the time available to the nursing professional for performing direct care, management, and educational activities and interventions [15].

All comments made by participants during the expert panel were recorded, and researchers, strategically distributed among the working groups, were responsible for compiling the experts’ main ideas. The researchers used a semi-structured interview guide to facilitate the discussion. In the fourth phase of the nominal group technique, all the central ideas identified in the speeches were presented and discussed collectively. Through deliberation and voting, the participants reached a consensus on the key indicators and conceptual definitions of the object of study. Prior to applying this technique, it should be noted that the researchers were trained in discourse analysis. This training allowed for a rigorous and structured interpretation of the interventions, thus facilitating the identification of common and divergent discursive patterns that enriched the consensus process.

After the first meeting with the first nominal group, all researchers met in a plenary session to re-listen to the participants’ speeches. This exercise allowed them to compare the information collected in the interview guides with the original interventions to ensure the accuracy and precision of the record. During this review, the researchers validated the points of convergence and divergence in the expressed opinions and refined the central elements obtained in the group session. This confirmed the derivation of the concept of nursing care burden.

The expert panels included diverse stakeholders, such as hospital general directors, nursing directors and frontline healthcare professionals. This was a deliberate strategy to ensure that the conceptual definition of nursing care burden was developed from a comprehensive, multi-level perspective. Each group made a unique contribution to identifying and refining key dimensions. Hospital general directors, for example, provided valuable insights into administrative and structural challenges, such as institutional policies and resource allocation issues that affect the delivery of nursing care. Nursing directors contributed their expertise in organizational workflows, staff management, and patient safety, emphasizing the operational implications of care burden at the service level. Meanwhile, frontline healthcare professionals, particularly staff nurses, offered grounded perspectives on the daily demands of direct care, the complexity of patient needs and the emotional and cognitive load involved in bedside practice. This multi-actor approach enabled the definition to encompass not only patient-centered care needs, but also systemic and managerial factors that influence nursing care delivery.

### 2.2. Phase 2: Design of the Measurement Scale

In this phase, the researchers defined the objective of the instrument: to evaluate the burden of nursing care by assessing the care needs of individuals admitted to inpatient services. Building on the results of the first phase and the definition of the instrument’s purpose, a second expert panel was convened, once again using the nominal group technique [38]. A group of registered nurses with graduate-level education and clinical experience in adult inpatient care within public and private health institutions of medium and high complexity, as well as directors of nursing departments, were invited to participate in the design of the instrument.

During the silent generation stage, the research project (including the previously developed conceptualization) was presented to the participants, along with two guiding questions intended to prompt individual reflection over approximately 15 min. The questions were: How should the burden of nursing care be assessed based on the agreed-upon conceptual definition? And what items should be included in such measurement instrument?

In the second step, involving a round of questions, participants’ responses were shared and read aloud, and all ideas were documented verbatim. As each response was presented, participants were invited to revisit the questions and elaborate on their contributions, with no time limit imposed.

The third step, clarification, involved academic discussion of the ideas gathered, with similar ideas being grouped together. In the final stage, voting and ranking were conducted, leading to the drafting of the instrument’s initial items by consensus.

The expertise of registered nurses with graduate-level education and clinical experience, as well as that of directors of nursing departments, played a complementary role in item development. The graduate-level nurses’ direct clinical experience was instrumental in identifying and articulating the specific, nuanced care needs of hospitalized patients. They drew on evidence-based practice and their understanding of patient–nurse interactions to do this. Their input ensured that the proposed items reflected the complexity of bedside care by integrating physical, emotional, and educational dimensions. In contrast, departmental directors contributed a broader managerial and systemic perspective, highlighting organizational factors, resource constraints, and workflow dynamics that influence the ability to meet these care needs. This dual perspective enabled the panel to operationalize the concept of nursing care burden by explicitly ‘placing care needs at the center of the measurement’: each item was reviewed and refined to ensure that it originated from a patient need rather than from task frequency or institutional demands alone. Through iterative discussions in nominal group sessions, the panel jointly considered clinical relevance and organizational feasibility, resulting in an item pool that captured the full scope of care—from direct patient support to indirect management activities—while maintaining patient needs as the foundation for measurement.

As a result of this second expert panel, it was concluded that the burden of nursing care should be assessed by operationalizing the previously developed concept, placing care needs at the center of the measurement. This process led to the drafting of 15 items addressing patterns of care needs, along with two additional items referring to the use of the Barthel Index and the Morse Fall Scale (MFS). The care needs assessed by the instrument include health promotion (item 1), nutrition (items 2, 3, 4), activity/rest (items 5, 6, 7), elimination (items 8, 9), perception/cognition (item 10), safety and security (items 11, 12, 13, 14, 15), comfort (item 16), and medication administration (item 17). Response options vary across items.

The decision to include the Barthel Index and the Morse Fall Scale (MFS) directly within the instrument was based on theoretical alignment and practical considerations. These internationally validated tools assess key functional domains—activities of daily living and fall risk—that directly and significantly impact nursing care burden. Functional dependency and fall risk are two of the most influential determinants of the amount and complexity of nursing care required, as they necessitate increased direct supervision, physical assistance, and preventive interventions, thereby intensifying the workload. Although these scales are usually used as separate assessments, incorporating them into the nursing care burden instrument ensures that these critical patient needs are systematically captured as part of the broader construct, maintaining a holistic, needs-based perspective. In this initial development phase, incorporating the Barthel Index and MFS avoids duplicating measurement efforts and leverages their strong psychometric foundations while enabling the exploration of their combined influence within a single conceptual framework. Future validation phases will enable the independent statistical evaluation of these components, ensuring their contribution to the total burden score is empirically supported and theoretically coherent.

The scale is structured into eight thematic domains—Health Promotion, Nutrition, Activity/Rest, Elimination/Exchange, Perception/Cognition, Safety/Protection, Comfort and Medication Administration—that group related care needs. Items within each domain are scored using different response formats depending on the nature of the activity being assessed. Most items use categorical response options that reflect gradations of need or dependency (e.g., no assistance, minimal assistance, moderate assistance, or high assistance), which are often based on recognized clinical scales such as the Barthel Index or the Braden Scale, or institutional risk assessment tools (e.g., fall risk assessment). Some domains, such as pain management, use intensity classifications derived from validated scales, such as the EVA (Visual Analogue Scale). The inclusion of various response formats rather than a uniform Likert scale was intentional, ensuring clinical relevance and facilitating integration with routinely collected patient assessment data. These categorical responses can be converted into numerical values, enabling the sum score of the instrument to reflect the overall nursing care burden while still allowing for analysis of specific domains.

The items developed enable the assessment of direct care and educational activities performed by nurses. However, it was agreed that the assessment of indirect care and care time would be addressed in a subsequent phase of the study, as these aspects may vary across institutions depending on the care models implemented in each context.

The Care Load Scale is designed for use by nursing professionals in hospital settings, particularly nurse managers or staff responsible for allocating patient care. The instrument is designed as a summative scale, whereby each item contributes to an overall score reflecting the burden of nursing care based on patient needs. It integrates dimensions of direct care, educational support, and management tasks, enabling a comprehensive evaluation of the complexity of patient care. Interpreting the scale enables healthcare teams to categorize patients according to the intensity of nursing care required, facilitating appropriate resource allocation, staff distribution, and quality monitoring. Higher scores indicate a greater care burden, informing managerial decisions regarding staff assignments, balancing workloads, and preventing adverse events related to nursing overload. Thus, the instrument provides nursing management with an evidence-based tool to support them in ensuring patient safety and optimizing care delivery.

Although the conceptual definition of nursing care burden proposed in this study considers the relationship between patient needs and the time required to provide care, including direct care, management activities, and educational activities, excluding the time component in this initial phase was a methodologically justified decision. This emerged during the second expert panel, where several participants emphasized the importance of measuring care time in order to standardize it alongside the other scale items. However, they also agreed that this measurement should be carried out at a later stage, once the instrument’s structure had been finalized.

As part of the third phase of the project and the construct validation process, it is planned that empirical measurements of the time required for nursing activities in different health institutions will be carried out. The aim is to standardize these times and establish their relationship with the categories identified in the developed scale.

It should be noted that accurate time estimation requires rigorous observational calibration, often involving time–motion studies or retrospective audits—methodologies that exceed the objectives and scope of this stage of instrument development. Therefore, a phased approach has been chosen, which will enable accurate assignment of care times to each activity category at a later stage while maintaining construct validity and consistency with the adopted theoretical framework.

### 2.3. Phase 3: Content Validity Through Expert Judgment

Before conducting the content validation, the face validity of the instrument was assessed. Face validity refers to the extent to which a measurement instrument appears, to experts or the target audience, to be appropriate and relevant for measuring the construct it is intended to assess [39]. For this purpose, a panel of six experts was convened, a number considered methodologically adequate in early stages of instrument development according to recommendations in the literature. The experts were selected using purposive sampling, ensuring diverse professional backgrounds directly related to the construct under study. The panel included nursing professionals with clinical experience in adult inpatient care, as well as academic researchers with expertise in instrument validation and psychometrics. All had at least five years of professional experience and held postgraduate qualifications (master’s or doctoral degrees). Experts were invited through formal email communication based on their research publications, academic affiliations, and recognized clinical expertise. Each expert evaluated the proposed items using a three-criteria matrix: comprehensibility or clarity (1 = completely comprehensible to 5 = incomprehensible), and relevance (1 = completely relevant to 5 = not relevant). Based on this evaluation, some items were revised to improve their comprehensibility prior to the content validation stage.

The validity of the content was assessed through expert judgement. The experts were selected using the snowball sampling technique. Initially, a systematic search of scientific articles was conducted in the LILACS databases and the Virtual Health Library (VHL) using the following descriptors: ‘Nursing Management Research’, ‘Nursing’, and ‘Psychometrics’. Based on the results, potential researchers were identified and their academic profiles and CVs reviewed. Their contact details were also obtained. Subsequently, a formal invitation to participate in the study was sent to them. Those who accepted were asked to suggest colleagues who met the following criteria:

− Be nursing professionals with postgraduate training (specialization, master’s degree or doctorate).
− Have clinical experience in hospital services.
− Have a background in scientific research.
− Be fluent in Spanish.

The selection and collection process took place over a two-month period, as defined by the researchers. Although the literature does not establish a fixed number of experts required for content validation, a robust panel of 55 expert judges was formed, strengthening the methodological rigor of the study.

Following the guidelines proposed by Waltz [37] and after reaching a consensus among the researchers, the evaluation criteria that each expert had to assess were defined as clarity, coherence, relevance, and sufficiency. The first three criteria were applied individually to each item, using categories and indicators based on the specialized literature (see Table 1) [37,40]. Sufficiency was evaluated globally for the entire instrument. Additionally, evaluators could include qualitative observations for each item and for the questionnaire as a whole.

The evaluation process was conducted anonymously: judges were not informed of the identities of the other participants. Based on their responses, the Content Validity Index (CVI) was calculated for each item (I-CVI) and the overall scale (S-CVI) [40].

If an item did not reach an acceptable CVI or if three or more judges considered it not relevant, it was excluded. Conversely, when items were deemed relevant and coherent, but two or more judges suggested improvements to enhance clarity, those suggestions were addressed, and the revised items were resubmitted to the expert group for review. Likewise, if four or more judges identified a lack of clarity in an item, it was modified accordingly [39,40,41].

All comments and suggestions regarding clarity, coherence, and relevance were compiled in an Excel matrix and subsequently processed using SPSS version 25. Based on consensus among the research team, the evaluators’ recommendations that were considered essential for improving the understanding of the items and the underlying construct were taken into account.

### 2.4. Statistical Analysis

Absolute and relative frequencies were used to describe the characteristics of the evaluators and their assessments of the instrument’s clarity, coherence, and relevance. The CVI was calculated at item and scale levels. This index was obtained by evaluating the relevance and representativeness of the items in relation to the definition of the concept. To calculate the item-level CVI (I-CVI), the number of experts who rated a given item with a score of 3 or 4 was divided by the total number of experts. To obtain the scale-level CVI (S-CVI), the sum of all I-CVIs was divided by the total number of items. A CVI equal to or greater than 0.90 is considered to indicate optimal content validity [40]. Additionally, Aiken’s V index was used to complement the assessment of content validity. This index was calculated using the following formula:

$$V=\frac{\bar{x}-1}{k}$$

where $\bar{x}$ is the mean of the ratings given by the judges, 1 is the lowest possible score given by the judges, and *k* is the range of possible scores (in this case, from 1 to 4).

A critical V value of 0.7 was set, based on the number of raters and the number of rating categories, and 95% confidence intervals were calculated. According to conventional interpretations, V values equal to or greater than 0.70 are considered acceptable, while values of 0.80 or higher are regarded as highly acceptable or very good [42].

Finally, Kendall’s W coefficient of concordance was calculated to assess the level of agreement among the evaluators. This non-parametric statistic tests the null hypothesis (H_0_) that there is no agreement among raters, meaning that rankings are random. A *p*-value < 0.05 was considered to indicate statistically significant concordance, thereby rejecting the null hypothesis and confirming that the level of agreement observed among the experts was unlikely due to chance [43,44].

## 3. Results

### 3.1. Face Vality

The face validity assessment revealed that the majority of the instrument’s items were rated as highly comprehensible by the six experts, with an overall mean score of 4.0 indicating a high level of comprehensibility. For example, the item on bowel elimination was initially worded as “Need for support with the patient’s bowel elimination: minimal, moderate, high” and was reworded to improve its clarity and specificity, finally becoming: “Need for support with the patient’s bowel elimination: requires minimal support (accompanying to the bathroom or providing a bedpan), moderate support (constipation or diarrhea), or high support (ostomies or rectal catheter).” This change allowed evaluators to better differentiate between categories of care.

Likewise, the wording of the item in the safety/protection domain “Need for care requirements for the patient’s wounds and injuries” was adjusted, eliminating the redundancy of the term “care requirements” and better specifying the types of wounds to facilitate clinical categorization. Another important change was made to one of the items in the comfort domain, “Pain management,” in which terms such as “mild, moderate, severe” were initially used and were replaced by “mild, moderate, and severe,” aligned with the VAS scale, thus improving consistency with standardized scales. These modifications were aimed at ensuring greater semantic clarity, clinical accuracy, and consistency with instruments validated in the scientific literature.

During this process, specific comments were identified, by consensus of the research team, that led to the modification of certain terms in some items to enhance their semantic clarity. Regarding coherence with the construct being measured, the items obtained an overall mean score of 4.0, suggesting that they were perceived as appropriate and conceptually aligned with the phenomenon under study. Similarly, the assessment of item relevance yielded a notably high result, with a mean score of 4.4, indicating that the experts considered the items to be representative and pertinent to the objective of the instrument.

### 3.2. Content Validity

The sociodemographic characteristics of the nurses who participated in the evaluation are presented in Table 2. Most of them were women residing in Colombia, with more than 10 years of professional experience and a specialization-level academic background. Most were actively working in clinical settings, particularly in adult inpatient care services. Participants’ ages ranged from 21 to 62 years, with a mean age of 36.

### 3.3. Content Validity Indices

Table 3 presents the frequency distribution of the relevance ratings assigned by the 55 expert judges. The I-CVIs for all items were higher than 0.80, indicating that each item demonstrated adequate content validity. Additionally, the S-CVI was 0.92, reaffirming the overall validity of the instrument.

As shown in Table 4, Aiken’s V values range from 0.84 to 0.94, indicating a high level of content validity across all items. All values fell within the 95% confidence interval, supporting the findings and suggesting strong consistency among the expert ratings.

### 3.4. Concordance of Judges’ Assessments

Table 5 shows the concordance indices for the dimensions of clarity, coherence, and relevance. The results indicate that Kendall’s W coefficients were all above 0.40 and statistically significant (*p* < 0.001), demonstrating a substantial level of agreement among the experts’ evaluations.

## 4. Discussion

This study was evaluated by a group of expert nurses with postgraduate education and extensive clinical experience in inpatient services, as well as in research, teaching, and management. These professional profiles align with the current trend of having specialists in clinical areas and the growing availability of specialists [45,46].

In nursing science, the CVI is a key tool in the validation of instruments. It enables the evaluation of each item individually, as well as the scale as a whole, allowing for the assessment of the validity of specific items while also obtaining a global validity estimate. One of its major disadvantages is that it does not account for chance agreement, which can lead to overestimation of consensus. For this reason, it is essential to complement CVI with statistical measures of consensus or concordance to strengthen the rigor and validity of the findings [47], as was conducted in the present research.

In this study, most evaluators rated the items as either fairly relevant or very relevant, with I-CVI values ranging from 0.89 to 0.95, and a S-CVI of 0.92. These findings are consistent with the recommendations in the literature, which suggest that CVI values above 0.80 are considered satisfactory for ensuring optimal content validity [47,48]. Likewise, the use of a large panel of experts reduces the risk of random bias in CVI calculations, thereby supporting a more robust and rigorous interpretation of the results.

In the present study, all Aiken’s V values were well above 0.7. In addition, all critical values were found to be within the range of the confidence intervals. These results indicate that all items should be retained, providing further confirmation of the instrument’s optimal content validity. Similar findings have been reported in previous studies that used Aiken’s V for content validation [49,50,51].

The results of the validation process also showed significant agreement in the evaluations of clarity, coherence, and relevance (*p* < 0.001). For clarity, the most frequent ratings were “clear but requires minor modifications” and “clear and requires no modifications,” indicating that the items were generally understandable. In terms of coherence, the most frequent rating across the 15 items was “coherent,” which, along with Kendall’s W statistic, supports the notion that the items are appropriate and conceptually aligned with the construct being measured. Regarding relevance, the predominant responses were “fairly relevant” and “very relevant,” which, together with the *p*-value < 0.001, indicates a high level of agreement among the judges regarding the representativeness and relevance of the items.

Kendall’s W coefficient is a commonly used indicator to assess the level of agreement among evaluators’ ratings. It should be noted that Kendall’s W does not measure agreement among judges, but rather the consistency in the ratings of items across judges. Evaluators may differ in their assessment of whether an item is clear, coherent, or relevant, but a significant Kendall’s W indicates a shared orientation in their ratings. The significance level associated with Kendall’s W helps confirm the robustness of the coefficient and lends confidence to the overall validation process. Generally, a *p*-value below 0.05 is considered sufficient to demonstrate a significant level of agreement and content validity [43,52].

This study makes a valuable contribution by developing and validating the apparent and content validity of the Nursing Care Burden Scale based on patients’ care needs. However, certain limitations must be acknowledged. Firstly, the current version of the instrument does not measure indirect care activities (e.g., documentation, coordination, and supply management) or care time, despite these being recognized components of nursing workload in the literature. This was a methodological decision, as estimating time requires rigorous observational calibration (e.g., time–motion studies), which will be addressed in a subsequent phase. Secondly, the validation process was conducted with a panel of experts and has not yet involved a field test with a representative patient sample, which restricts the generalizability of the results.

Future research should focus on integrating indirect care and standardized care time measurements to strengthen the scale’s construct validity and enhance its ability to reflect the multidimensional nature of nursing care burden. Additionally, it will be important to evaluate the applicability of the scale in various clinical settings, such as medical–surgical units, intensive care units, and long-term care facilities, and to determine its usefulness in care planning and resource allocation. Such studies could assess how the scale helps nurse managers to identify high-burden scenarios, prioritize interventions, optimize staffing, and ultimately improve patient safety and the quality of care.

All expert panels in this study were composed exclusively of professionals from Colombian healthcare institutions. While this approach ensured contextual relevance and alignment with the local healthcare system, it may limit the generalizability of the findings to other national or international settings with different organizational structures, cultural contexts, and nursing practice environments. To strengthen the external validity and broaden the applicability of the instrument, future validation studies should incorporate participants from a wider range of institutions, both within Colombia and across different countries, allowing for cross-cultural comparisons and adaptation to diverse healthcare contexts.

## 5. Conclusions

The preliminary version of the Inpatient Nursing Care Burden Scale demonstrated strong content validity, supporting its potential utility in assessing the burden of nursing care based on patients’ individual care needs. However, a third phase of the research project is planned to strengthen the psychometric validation of the instrument. This phase will involve field testing the scale with a sufficiently large and diverse sample of hospitalized patients to conduct an Exploratory Factor Analysis (EFA) and, subsequently, a Confirmatory Factor Analysis (CFA). These analyses aim to assess the construct validity of the instrument and identify its underlying factor structure. In addition, internal consistency reliability will be evaluated using appropriate statistical techniques (e.g., Cronbach’s alpha). Conducting these procedures is essential to ensure both the internal and external validity of the instrument and to confirm its applicability across different clinical contexts and patient populations.

## Figures and Tables

**Table 1:** Evaluation criteria for content validation through expert judgement.

Evaluation Criteria	Score and Evaluation Categories	Indicator
Clarity: Ability to identify and understand the intent of the item	1. Unclear	The wording, grammar, or spelling prevents the reader from comprehending the main idea of the item, making it difficult to interpret.
	2. Not clear and requires substantial modifications	The main idea of the item is not understood. Significant changes in wording, grammar, or spelling are needed to make it understandable to the reader.
	3. Clear but requires substantial modifications	The main idea of the item is understood, but multiple changes in wording, grammar, or spelling are needed to improve clarity for the reader.
	4. Clear but requires minor modifications	The main idea of the item is understood, but one or two words need to be adjusted to improve clarity, grammar, or spelling, making it more understandable to the reader.
	5. Clear and requires no modifications	The main idea is clearly conveyed, and the wording, grammar, and spelling allow for full comprehension by the reader.
Coherence: Logical alignment with the construct being measured.	1. Not coherent	The item lacks a logical and coherent relationship with the dimension it is intended to measure, which compromises its relevance in the context of the evaluation.
	2. Slightly coherent	The item presents a weak or limited logical relationship with the dimension it aims to measure, suggesting an insufficient or irrelevant contribution to the construct.
	3. Moderately coherent	The item demonstrates a moderate logical relationship with the dimension. While potentially useful, it may require refinement to enhance accuracy.
	4. Highly coherent	The item shows a logical and coherent relationship with the dimension. While potentially useful, minor adjustments may still be needed to improve accuracy.
	5. Coherent	The item has a sound and coherent relationship with the proposed dimension, supporting its inclusion and making a significant contribution to measuring the construct.
Relevance: Representativeness and importance of the item in the instrument	1. Irrelevant	The item can be removed without affecting the measurement of the dimension.
	2. Not very relevant	The item can be modified without significantly impacting the measurement of the dimension.
	3. Somewhat relevant	The item holds limited relevance; its content may be captured by another item.
	4. Fairly relevant	The item is moderately important in measuring the dimension.
	5. Very relevant	The item is relevant and should be included.

**Table 2:** Sociodemographic characteristics of the evaluating judges.

Variable	Frequency	Relative Frequency
Sex		
Man	22	40.00%
Woman	33	60.00%
Years of professional experience		
1–2 years	8	14.50%
3–4 years	5	9.10%
5–6 years	3	5.50%
6–7 years	3	5.50%
7–8 years	6	10.90%
9–10 years	5	9.10%
More than 10 years	25	45.50%
Educational level attained		
Specialization	31	56.40%
Master’s degree	23	41.80%
PhD	1	1.80%
Faculty category		
Full professor	7	12.70%
Assistant professor	9	16.40%
Associate professor	5	9.10%
Instructor	4	7.30%
Not a faculty member	30	54.50%

**Table 3:** Frequency of relevance ratings and Content Validity Index for each item.

Items	Frequency of Relevance Ratings					I-CVI ^1^
	Irrelevant	Not Very Relevant	Somewhat Relevant	Fairly Relevant	Very Relevant	
Item 1	1	0	2	12	40	0.95
Item 2	0	1	2	15	37	0.95
Item 3	0	1	4	16	34	0.93
Item 4	0	1	3	11	40	0.93
Item 5	0	0	6	18	31	0.89
Item 6	0	0	2	13	40	0.96
Item 7	0	0	5	12	38	0.91
Item 8	0	0	4	14	37	0.93
Item 9	0	0	5	14	36	0.91
Item 10	0	0	3	11	41	0.95
Item 11	0	0	3	7	45	0.95
Item 12	0	0	5	9	41	0.91
Item 13	0	1	3	15	36	0.93
Item 14	0	0	4	10	41	0.93
Item 15	0	1	5	8	41	0.89

^1^ I-CVI: Item-level Content Validity Index.

**Table 4:** Aiken’s V for assessment of each item by expert judges.

Item	Criterion	Aiken’s V	95% CI
1. Patient’s and family’s need for education.	Clarity	0.84	0.78–0.87
	Coherence	0.84	0.78–0.88
	Relevance	0.91	0.86–0.94
2. Patient’s need for enteral nutrition care	Clarity	0.87	0.82–0.91
	Coherence	0.85	0.79–0.89
	Relevance	0.90	0.85–0.93
3. Patient’s need for parenteral nutrition care	Clarity	0.90	0.84–0.92
	Coherence	0.88	0.82–0.91
	Relevance	0.88	0.82–0.91
4. Patient’s need for glycemic control.	Clarity	0.89	0.84–0.92
	Coherence	0.90	0.84–0.92
	Relevance	0.91	0.86–0.94
5. Patient’s need for supplemental oxygen.	Clarity	0.89	0.83–0.92
	Coherence	0.86	0.80–0.89
	Relevance	0.86	0.81–0.90
6. Patient’s need for follow-up to clinical stability.	Clarity	0.85	0.79–0.89
	Coherence	0.86	0.81–0.90
	Relevance	0.92	0.87–0.95
7. Patient’s need for urinary-elimination-mediating devices.	Clarity	0.92	0.87–0.94
	Coherence	0.90	0.85–0.93
	Relevance	0.90	0.85–0.93
8. Patient’s need for bowel elimination support.	Clarity	0.88	0.83–0.91
	Coherence	0.88	0.83–0.91
	Relevance	0.90	0.85–0.93
9. Patient’s need for support based on patient’s level of restlessness	Clarity	0.85	0.80–0.89
	Coherence	0.86	0.81–0.90
	Relevance	0.89	0.84–0.92
10. Patient’s need for care according to fall risk.	Clarity	0.88	0.83–0.91
	Coherence	0.88	0.82–0.91
	Relevance	0.92	0.87–0.95
11. Patient’s need for wound and injury care.	Clarity	0.91	0.86–0.94
	Coherence	0.89	0.83–0.92
	Relevance	0.94	0.90–0.96
12. Patient’s need for vascular access	Clarity	0.86	0.80–0.89
	Coherence	0.89	0.83–0.92
	Relevance	0.91	0.86–0.94
13. Patient’s need for isolation measures.	Clarity	0.88	0.83–0.91
	Coherence	0.87	0.81–0.90
	Relevance	0.89	0.84–0.92
14. Need for pain management.	Clarity	0.86	0.80–0.89
	Coherence	0.88	0.82–0.91
	Relevance	0.92	0.87–0.94
15. Need for medication administration.	Clarity	0.87	0.82–0.91
	Coherence	0.85	0.79–0.89
	Relevance	0.90	0.85–0.93

**Table 5:** Concordance of judges’ assessments.

Indices	Kendall’s W Statistic
Clarity	0.54 ***
Coherence	0.70 ***
Relevance	0.73 ***

*** *p* < 0.001.

## Data Availability

The data that support the findings of this study are available on request from the corresponding author.

## Public Involvement Statement

No public involvement in any aspect of this research.

## Guidelines and Standards Statement

This manuscript was written in accordance with the COSMIN Study Design Checklist for Instrument Validity Research.

## Abbreviations

The following abbreviations are used in this manuscript:

CVI	Content Validity Index
I-CVI	Item-level Content Validity Index
S-CVI	Scale-level Content Validity Index
MFS	Morse Fall Scale
SPSS	Statistical Package for the Social Sciences
CI	Confidence Interval
PhD	Doctor of Philosophy
